# The relationship between miRNA-210 and SCN1B in fetal rats with hypoxic-ischemic brain injury

**DOI:** 10.1042/BSR20222016

**Published:** 2023-01-16

**Authors:** Hisham Al-Ward, Ning Liu, Moussa Omorou, Yiwei Huang, Wei Chen, Chun-Yang Liu, Shaochun Lv, Abduh Murshed, Fahmi Shaher, Yao Li, Yuxuan Zhang, Linxia Lu, Wenxia Gao, Yi Eve Sun, Hui Xu

**Affiliations:** 1 Shanghai Institute of Stem Cell Research and Clinical Translation, Shanghai East Hospital, School of Medicine, Tongji University, Shanghai 200065, China; 2 Department of Biochemistry and Molecular Biology, School of Basic Medical Sciences, Jiamusi University, Jiamusi 154007, China; 3 Department of Rehabilitation therapy, School of Medicine, Ankang University, Ankang 725000, China; 4 Provincial Key Laboratory of Microecology Immune Regulation Network and Related Diseases, Basic Medical College, Jiamusi University; 5 Department of Clinical Laboratory, Shanghai 10^th^ People’s Hospital of Tongji University, Shanghai 200072, China; 6 Department of Pathology and Pathophysiology, School of Basic Medical Sciences, Jiamusi University, Jiamusi 154007, China; 7 Department of Clinical Laboratory Diagnosis, School of Basic Medical Sciences, Jiamusi University, Jiamusi 154007, China

**Keywords:** Hypoxia, Hypoxic ischemic encephalopathy, miR-210, miRNA, SCN1B, Voltage Gated Sodium Channels

## Abstract

Hypoxic-ischemic brain injury contributes to major neurodevelopmental disorders and is one of the leading causes of seizures, which substantially results in neurodevelopmental impairments with long-lasting outcomes and is one of the main causes of death in neonates. We aimed to investigate the correlation between miRNA-210 and SCN1B, a voltage-gated sodium channel gene, in brain tissue of fetal rats with hypoxic-ischemic brain injury. We found that after 10 min of hypoxia-ischemia, all reperfusion groups showed different degrees of damage. The degree of the injury increased in all the groups after 30 min of hypoxia-ischemia. Those changes include changes in the pericellular lumen, capillaries in the cortex, erythrocytes, enlarged pericellular lumen, the enlarged pericapillary lumen in the cortex, edema around glial cells, enlarged gap to form multiple necrotic foci, deformation of neurons, and loss of cell structure. The expression levels of HIF-1α, miRNA-210, and HIF-1α mRNA were higher in the hypoxic-ischemic groups than that in the control groups, among which the expression levels in the severe group were higher than that in mild group. SCN1B is down-regulated in both the mild and severe groups, and the lowest level was found at 30 min after hypoxia in both groups. MiRNA-210 plays a role in the development of hypoxic-ischemic encephalopathy (HIE) by regulating the expression changes of SCN1B. The brain tissue of fetal rats in the hypoxic-ischemic animal model showed pathological changes of brain injury.

## Introduction

Neonatal hypoxic-ischemic encephalopathy (HIE) is a serious disease that is a complication of perinatal neonatal asphyxia. HIE causes neurodevelopmental disorders with varying degrees of sequelae after prolonged onset, such as cerebral palsy, learning disabilities, mental retardation, and seizures [1]. HIE mainly causes neuronal damage and is a serious threat to the life of the newborns [2,3].

HIF-1α is involved in pathophysiological processes such as cerebrovascular disease, neurological injury, and myocardial ischemia. It was found that HIF-1α has a cerebral protective effect in cerebral hypoxic-ischemic injury, and a decrease in oxygen level increases HIF-1α protein and gene expression activity. Sodium channels are widely distributed in human tissues and play an important role in maintaining normal physiological functions. The current generated by sodium channels constitutes the rising phase of action potentials, and sodium channel proteins are composed of α and β subunits together. There are ten α subunits and four β subunits in sodium channels. SCN1B gene encodes β1 multifunctional auxiliary subunit of sodium channels, which has various functions, including regulating the opening and closing of Na^+^ channels, regulating their expression level in the cell membrane, interacting with the extracellular matrix as intercellular adhesion molecules, and participating in regulating cell migration and aggregation [4].

MicroRNAs (miRNAs) are a class of highly conserved single-stranded RNAs, consisting of about 22 nucleotides in length, that play an essential regulatory role in the developmental and metabolic processes of the body. Many miRNAs exist in the central nervous system, which is closely related to the development, differentiation, and physiological functions of nerve cells and play an important regulatory role in neuropathy and dysfunction after cerebral hypoxia-ischemia. Specific miRNAs can affect the expression of their target genes after hypoxia-ischemia through their own level changes and participate in the regulation of neuroprotective mechanisms and apoptotic regeneration mechanisms [5]. Abnormal miRNA expression has been observed in a variety of diseases [6]. MiRNAs represent approximately 1–2% of the transcriptome of eukaryotic cells and play a crucial roles [7], and these molecules also stimulate post-transcriptional regulation of gene expression through direct or indirect mechanisms [8].

HIF-1α binds to hypoxia response elements (HREs) at the miRNA-210 promoter closest to the transcription start site [15]. Comparison of miRNA-210 core promoters in different organisms indicates that the HRE region is highly conserved and that Hypoxia is critical for the regulation of miRNA-210 expression across multiple species. The induction of miRNA-210 under hypoxic conditions in rats is dependent on HIF-1α [9].

In recent years, the role of HIF-1α in the central nervous system has been continuously recognized. More and more studies have shown that HIF-1α has a cerebral protective effect in cerebral hypoxic-ischemic injury. Current studies have found that HIF-1α is involved in pathophysiological processes such as cerebrovascular disease, neurological injury, tumors, myocardial ischemia, pulmonary hypertension, preeclampsia, and fetal growth retardation in utero. MiRNA-210 expression is up-regulated, while HIF-1α content is also increased, indicating that hypoxia-ischemia causes an increase in HIF-1α content, HIF-1α induces miRNA-210 expression. The increase of miRNA-210 expression would promote the repair of brain tissue damage.

The SCN1B gene encodes the β1 multifunctional auxiliary subunit of the sodium channel, which is involved in the regulation of Na^+^ channels and in the regulation of Na^+^ channel expression levels [10]. This gene also encodes the β 1B variant, formerly known as β 1A. The gene is located at approximately 9.0 kb on chromosome 19 at position 13.11 and consists of six exons (>136, 166, 240, 141, 71, and >641 bp) and five introns (1.67, 1.80, 5.38, 0.38, and 0.09 kb) [11]. In humans, studies on tissue distribution have shown that the β1B subunit is expressed mainly in skeletal muscle, spinal cord, and brain [12]. Human SCN1B mutations have been associated with generalized febrile seizures (GEFS+) and Dravet syndrome (DS) [13].

Over the past few decades, great progress has been made in the treatment of neonatal disorders and the level of care for HIE has improved. At the same time, as the level of perinatal care has improved, the survival rate of low gestational age infants has increased, leading to an increase in the number of high-risk fetuses surviving and, consequently, an increase in the incidence of HIE. By fully understanding the functions and regulatory mechanisms of specific miRNAs in the brain, it will help to provide new strategies for the prevention, diagnosis, and treatment of HIE from the perspective of gene expression regulation. While the sodium channels under the hypoxic conditions are widely investigated, the role of miRNA regulated by hypoxia in these channels still largely not fully understood especially β subunit genes. In the present study, we aimed to investigate the correlation between miR-210 and SCN1B under the hypoxic condition.

## Animal grouping and treatment

### Animal grouping

Rats were assigned to five different cages in a ratio of 2:1, and female rats were examined for pregnancy every morning by microscope and measuring their weights; the pregnant rat kept in a separate cages. Sprague Dawley (SD) female rats at 19 days of pregnancy were selected and divided into normal and model groups.

(1) Normal group (control): Samples (brain and blood) were collected on day 19 of gestation without any treatment. (2) In the model group, pregnant rats were anesthetized with xylazine on day 19 of gestation, and a median incision exposed the uterine horn; the branch vessels entering each placenta were isolated and clamped with noninvasive vascular clamps to create a mild hypoxic-ischemic brain injury model (clamped for 10 min) and a severe hypoxic-ischemic brain injury model (clamped for 30 min). Then, the vascular clamps were released to restore blood flow and reperfusion for 10, 30 min, 1, and 3 h, respectively. The model groups were divided into eight groups, i.e., 10, 30 min, 1, and 3 h for mild hypoxic-ischemic reperfusion; 10, 30 min, 1 and 3 h for severe hypoxic-ischemic reperfusion. After reaching the specified time, the fetal rats were directly taken from the placenta, and the brain and blood were collected for relevant tests. At the end of the experiment, all animals were anesthetized by intraperitoneal pentobarbital sodium and sacrificed by CO_2_ asphyxiation. All animal experiments took place at the school of basic medical sciences, Jiamusi University. The study approved by the Research Ethics Committee of Jiamusi University (JD10021).

### Animal treatment and sampling

Brain samples were taken from each experimental group, samples were washed with diethyl pyrocarbonate (DEPC-treated) saline, wrapped in tinfoil, and then put into liquid nitrogen for freezing and storage for western blot experiments. Samples from each group were preserved in 4% paraformaldehyde, fixed for 24 h, and then stored in 4°C refrigerators for haemotoxylin and eosin (H&E) staining. For the PCR test, the fetal rat brain tissue samples were stored at –80°C without any treatment. Serum samples were collected and stored at –80°C for the enzyme-linked immunosorbent assay (ELISA) test.

## Histomorphological observation of brain tissue of fetal rats

The fetal rat brain tissue was fixed with 4% paraformaldehyde for 24 h, then embedded in paraffin, sectioned (thickness: 4 μm) onto a glass slide, and then dewaxed with toluene stained with H&E. The steps of H&E staining were as follows:

Slides were dried in an oven at 60°C for 30 min and dewaxed in xylene I, II, and III for 10 min each. Then, washed in anhydrous alcohol I and II for 5 min each; slides were immersed in 95%, 80%, and 70% graded alcohol for 3 min each.

The slides were washed with distilled water to remove the alcohol and hydrate; immersed in hematoxylin stain for 10 min; then rinsed with running water for 2 min. Samples were kept in 0.5% hydrochloric acid in alcohol for 30 s; washed with running water for 2 min, with 0.1% ammonia for about 30 s, and with water for 30 s, then transferred to 0.5% EOSIN solution and kept for 2 min.

Slides were immersed in distilled water for a few seconds; kept in 70%, 80%, and 95% gradient alcohol for 3 min each; then immersed in anhydrous alcohol I and II for 5 min each (dehydration). All samples submerged in xylene I and II for 10 min each (transparency). The neutral resin was used to seal the slices, and the pathological changes of the fetal rat brain were observed under the light microscope.

## ELISA for the determination of HIF-1α in fetal rats

ELISA was used to quantify the level of HIF-1α in fetal rat serum. The protein levels of HIF-1α in the samples were measured using human ELISA kits according to the manufacturer’s instructions (Shanghai Enzyme-Linked Biotechnology Co., Shanghai, China).

## q-RT-PCR to detect the expression level of fetal rat brain miRNA-210, and HIF-1α mRNA

Based on the HIF-1α and β-actin mRNA sequences provided by Gene Bank, primer sequences were designed using Primer Premier 5.0 software, and no significant similarity with other genes was confirmed by the Blast test.

The primers were designed and synthesized by Bao Biological Engineering (Dalian) Co. Total RNA was extracted from fetal rat brain tissue by using the miRamp kit (TianGenBiotech Co., Beijing, China). Then, cDNA was synthesized using the miRcute Plus miRNA First-Standard cDNA kit (TianGenBiotech Co., Beijing, China). SYBR Green miRcute Plus miRNA qPCR Kit (TianGenBiotech Co., Beijing, China) was used to perform real-time PCR. The procedure was done following the manufacture’s guidelines. The levels of PCR products were normalized with U6. The relative quantitative level of miRNA-210 was determined by means of the RQ = 2^−ΔΔCt^ method.

## Protein blot (western blot) analysis of HIF-1α and SCN1B expression in fetal rat brain tissues

Protein blotting (western blots) was applied to detect the expression levels of HIF-1α and SCN1B proteins in the brain tissues of fetal rats in each group, and the differences in expression levels between the groups were compared.

### Protein lysis

Fetal rat brain tissues were lysed with RIPA buffer, lysed at 4°C, then centrifuged at 12000 ***g***, 4°C for 20 min, the supernatant was taken and put in liquid nitrogen tank or –80°C refrigerator for protein determination before each electrophoresis. The protein was assessed by BCA Protein Assay Kit. Then, the SDS-polyacrylamide gel electrophoresis was performed. The primary antibodies used are HIF-1α Ab109 ##440, SCN1B, both are from Affinity Biosciences, China.

The SuperSignal™ West Pico Chemiluminescent Substrate (Thermo Fisher Scientific, Waltham, MA, U.S.A.) was used to visualize proteins in the membrane. Protein band intensities were assessed using LabWork 3.0 (UVP Inc., Upland, CA), and protein band densitometry was performed using ImagePro Plus 6.0 imaging software.

### Statistical analysis methods

The measurement data conforming to the normal distribution were expressed as mean ± standard deviation, we compared the expression levels in the case groups with the control groups, and the differences between groups were processed by one-way ANOVA, with *P*<0.05 as statistical difference and *P*<0.01 as significant difference. Western blot experimental data were expressed as mean ± standard error main value (x ± SE), and all quantitative analyses were performed by Tukey’s post-hoc analysis using the ANOVA one-way method, with *P*<0.05 as statistical difference and *P*<0.01 as significant difference, and statistical analysis of all experimental data was performed with GraphPad Prism 8 program (San Diego, CA, U.S.A.).

## Results

### Analysis of miRNA-210-targeting regulation of SCN1B

The miRNAs with potential regulatory effects on SCN1B were analyzed by TargetScan, a bioinformatics website, and combined with the group’s previous research, the analysis showed that miRNA-210 has potential regulatory effects on SCN1B, and SCN1B is a target gene of miRNA-210.

The base sequence of miRNA-210: CCAGGCGCAGGGCAGCCCCUGCCCACCGCACACUG, and the base sequence of the 3′-UTR of the mRNA of SCN1B:

GGUCCGUGUCUAGUCGGCGACAGUGUG CGUGUCAC. MiRNA- 210 binds to the 3′-UTR of the mRNA of SCN1B by noncompletely complementary base-pairing binding.

### Histopathological changes in the brain of fetal rats

#### Morphological observation of brain tissue in the control group

**Figure 1:**
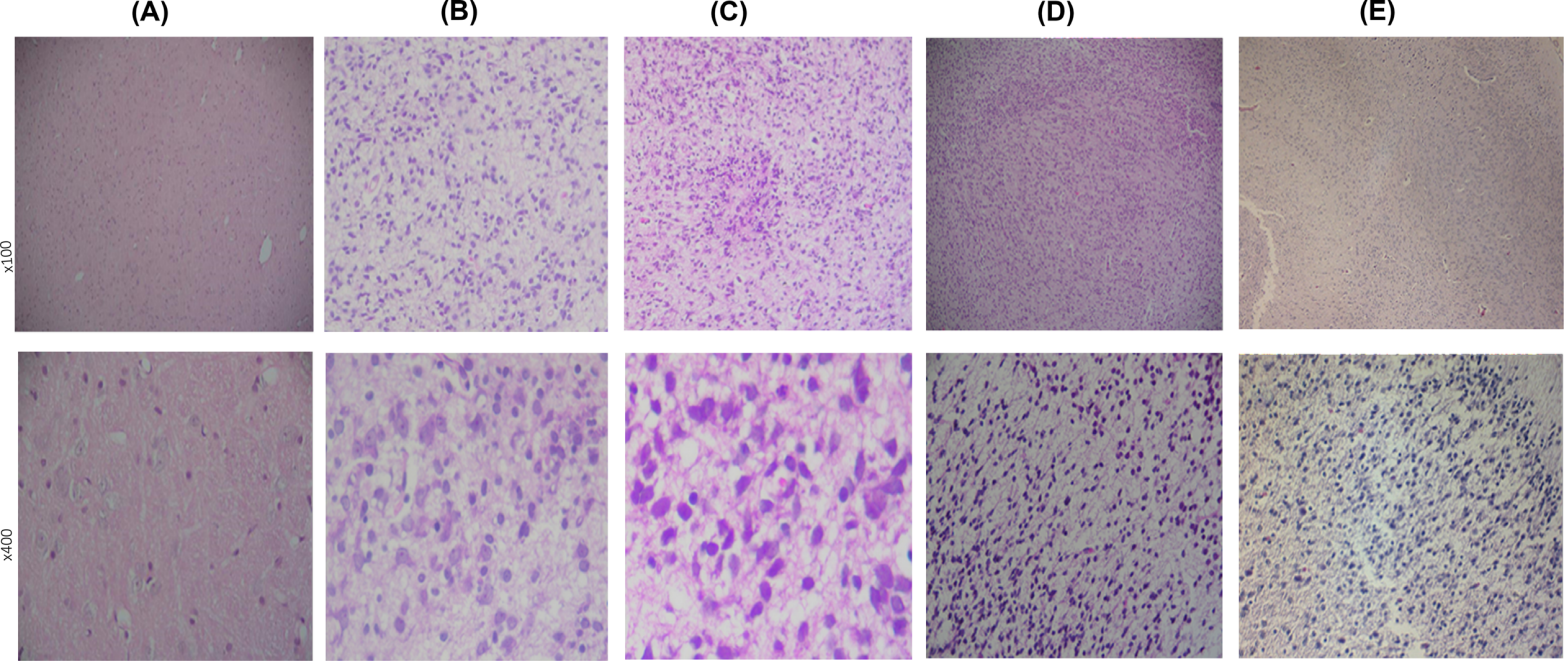
Histopathological changes among groups in the mild hypoxic ischemic group No changes in the control group. Several changes are obvious in the hypoxic-ischemic groups, changes varies depending on the hypoxia severity mild group: (**A**) = control; (**B**) = reperfusion 10 min, changes in the microglia, glial cells, neurons, and cytoplasm occured; (**C**) = reperfusion 30 min, microglia cells are highly affected by the severity of the hypoxic insult, necrosis, and lose of structure are shown; (**D**) = reperfusion 1 h; (**E**) = reperfusion for 3 h. In both groups (**C,D**), the severity of the hypoxic insult led to major effects on the cell including edema.

There were no obvious changes in the morphology of brain tissue of fetal rats in the control group, all of which showed clear cortical and white matter structures; the neuronal layers in the cortical and molecular layers were clear, inner and outer granular layers, pyramidal cell layer, polymorphic cell layer, and ganglion cell layer could also be observed; neurons were filled with glial cells in the middle; brain tissue of fetal rats was rich in capillaries, and red blood cells were seen in the capillary lumen (Figures 1A ).

**Figure 2:**
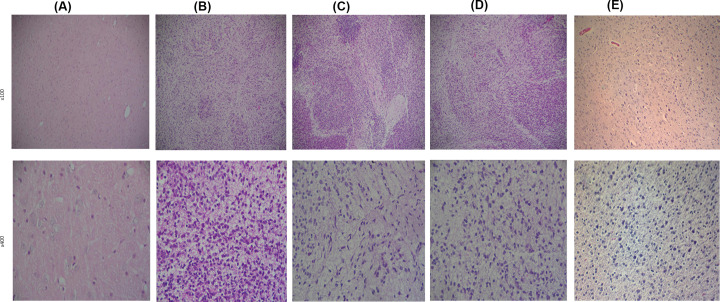
Histopathological changes among groups in sever hypoxic ischemic group (**A**) = reperfusion 10 min, changes in the microglia, glial cells, neurons, and cytoplasm occurred. Microglia showed diffuse hyperplasia with pericellular edema; (**B**) = reperfusion 30 min, microglia cells are highly affected by the severity of the hypoxic insult, necrosis and lose of structure are shown; Glial cells showed diffuse proliferation, neurons were affected by the hypoxic insult; (**C**) = reperfusion 1 h; (**D**) = reperfusion for 3 h. in both groups (**C,D**), the severity of the hypoxic insult led to major effects on the cells, the periglial cells were edematous and the gap increased to form multiple foci of necrosis.

#### Histopathological changes of brain of fetal rats in hypoxic-ischemic group

In mild hypoxic ischemic group, after 10 min of reperfusion, microglia showed diffuse hyperplasia, glial cells were swollen, nuclei were obviously enlarged, and gaps were enlarged to form multiple edema foci. Some neurons were swollen and deformed, with vacuolation of cytoplasm and loss of structure (Figure 1B).

Microglia were diffusely proliferated with focal hyperplasia and microglia nodules were seen in the group with reperfusion for 30 min. The periglial cells were edematous and the gap was enlarged to form multiple foci of necrosis. The neurons were deformed, with crumpled cytosol, nucleus consolidation, necrosis, and loss of structure (Figure 1C).

Microglia were diffusely proliferating with localized focal hyperplasia in the group with reperfusion for 1 h. The periglial cells were edematous and the gap increased to form multiple foci of necrosis (Figure 1D). Microglia were diffusely proliferating with localized focal hyperplasia in the group with reperfusion for 3 h. Glial cells were swollen, with markedly enlarged nuclei and periglial edema (Figure 1E).

In severe hypoxic-Ischemic group, microglia showed diffuse hyperplasia with localized focal hyperplasia, edema and increased gap with focal necrosis after reperfusion for 10 min (Figure 2A).

In the severe group of hypoxia-ischemia with 30 min of reperfusion, neuronal cells degenerated and necrotic, glial cells showed diffuse proliferation with focal hyperplasia, and microglia aggregated into clusters and formed microglia nodules. The interstitial spaces of glial cells increased to form multiple foci of necrosis. The neurons were degenerated and necrotic, with crumpled cytosol, nucleus consolidation, necrosis, and loss of structure (Figure 2B). In the group with reperfusion for 1 h, glial cells showed diffuse hyperplasia with focal hyperplasia, and microglia aggregated into clusters to form microglial nodules. The periglial cells were edematous and the gap increased to form multiple foci of necrosis. The neurons are deformed and the cell structure is lost (Figure 2C). In the group with reperfusion for 3 h, glial cells showed diffuse hyperplasia with focal hyperplasia, and microglia aggregated into clusters to form microglial nodules. The glial cell gap increased to form multiple foci of necrosis (Figure 2D).

### ELISA method to detect blood HIF-1α content

**Figure 3:**
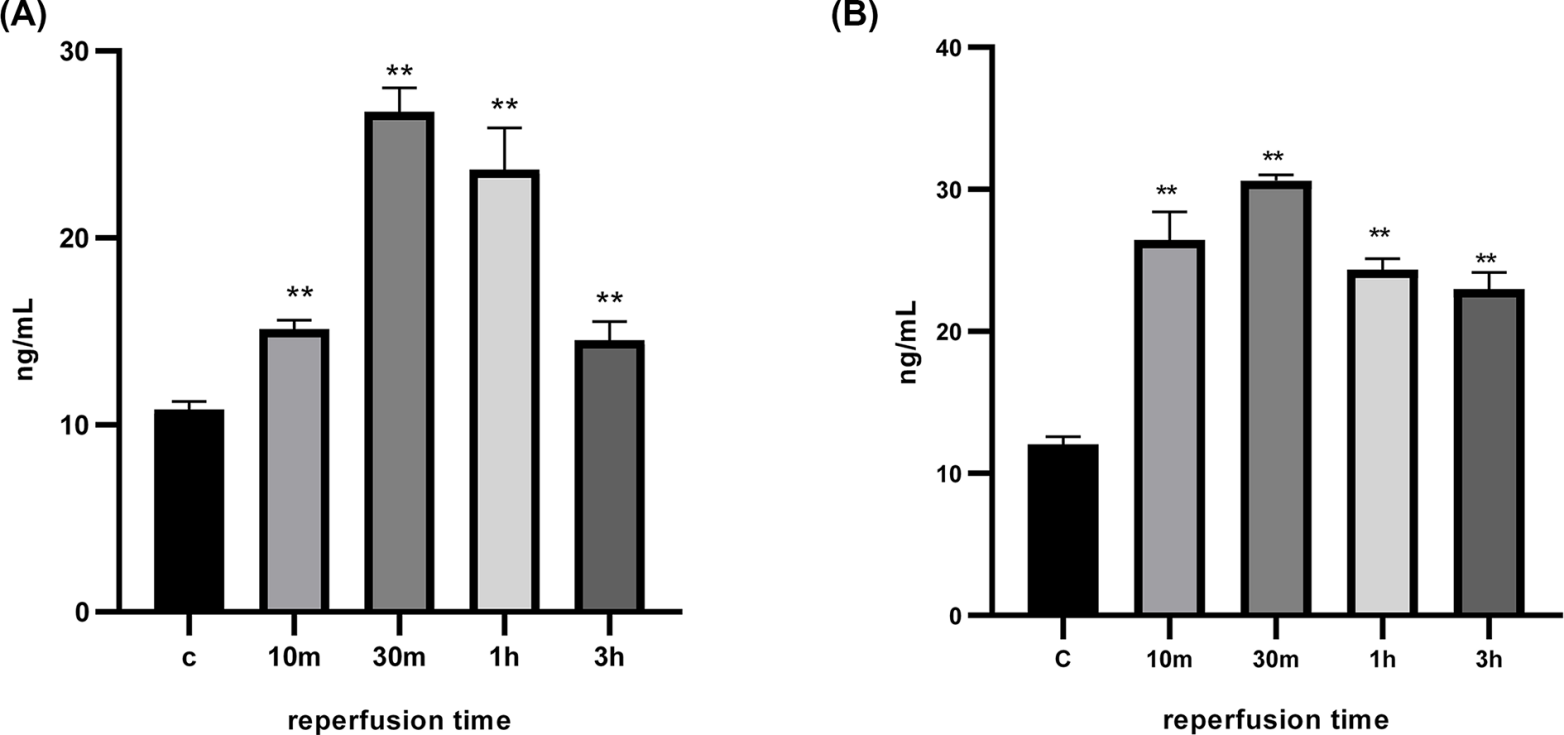
Expression levels of HIF-1α in rats with mild and severe hypoxia ischemia (**A**) Serum HIF-1α level of fetal rats in each group of mild hypoxia-ischemia, F (DFn, DFd) are F (4, 35) = 223.8. (**B**) Serum HIF-1α level of fetal rats in each group of severe hypoxia-ischemia. F (DFn, DFd) are F (4, 35) = 37.81. **The differences in both mild and severe groups are statistically significant (*P*<0.01) when compared with the control group.

Compared with the control group, the HIF-1α values were significantly higher in each group at 10 and 30 min of hypoxia-ischemia in fetal rats, and the highest at 30 min of reperfusion, and the differences were statistically significant (*P*<0.01), (Figure 3).

### q-RT-PCR

In the present study, ten samples (brain tissues) of fetal rats were selected from the control group, and ten samples for each mild hypoxia (reperfusion for 10, 30 min, 1, 3 h) group and ten samples for each of sever hypoxia (reperfusion for 10, 30 min, 1, 3 h) group.

The miRNA was extracted from fetal rat brain tissue samples, and the RNA concentration and purity were determined using a nucleic acid quantification instrument. The optical density A260/A280 were all between 1.8 and 2.0, indicating no degradation of the RNA and good quality.

After the miRNA was reverse transcribed to generate cDNA, q-RT-PCR was performed for each group of samples, and no spurious peaks were seen in the solubility curve analysis, indicating a single amplification product and no nonspecific amplification. The amplification curves showed that all samples had entered the amplification plateau phase, indicating that the reaction conditions were set accurately.

**Figure 4:**
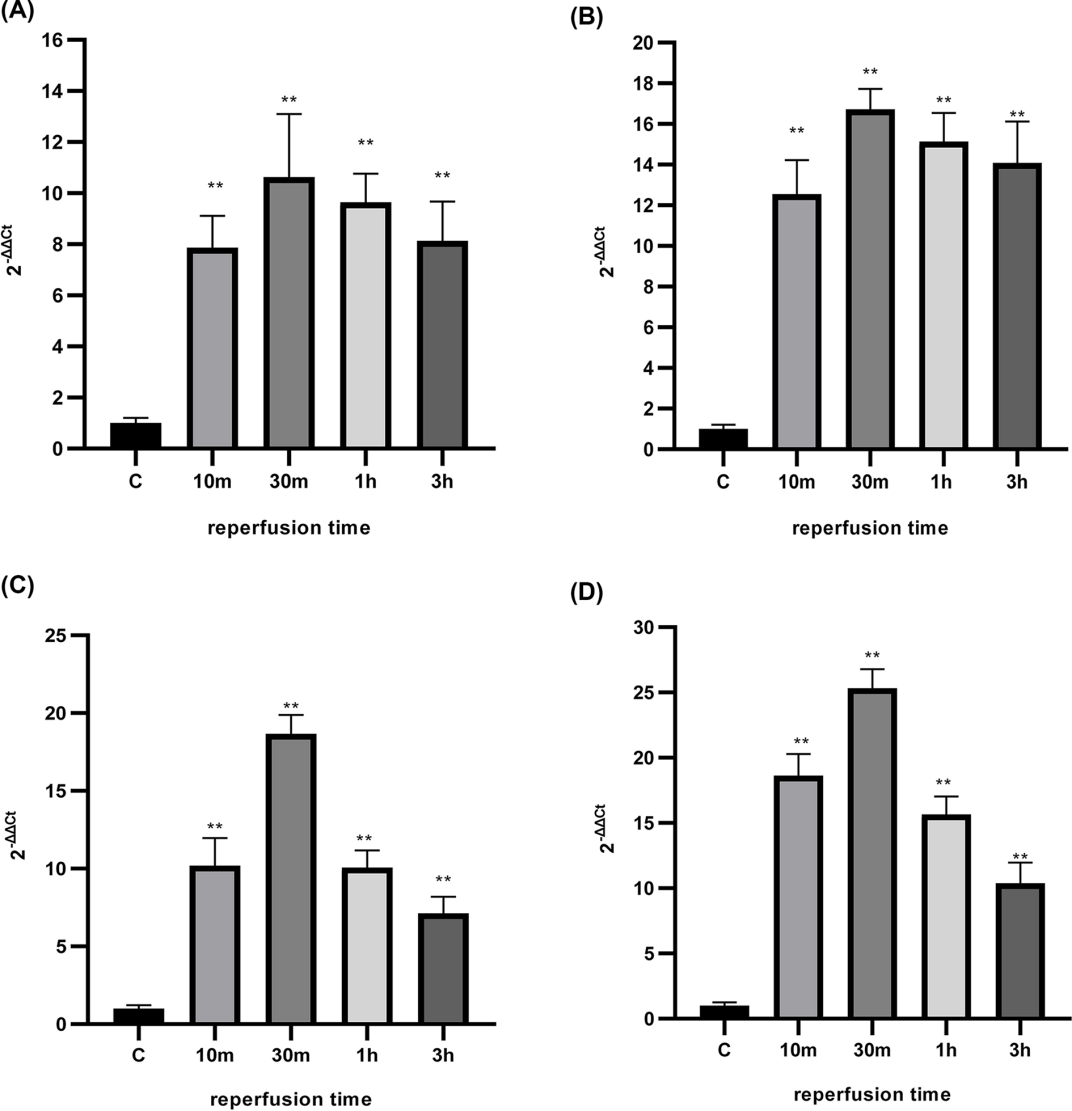
Expression levels of miR-210 in rats with and without (the control) hypoxia-ischemia brain damage (**A**) Expression of miRNA-210 in samples from each group with mild hypoxia-ischemia, F (DFn, DFd) are F (4, 35) = 50.60. (**B**) Expression of miRNA-210 in samples from each group with severe of hypoxia-ischemia, F (DFn, DFd) are F (4, 35) = 159.2. (**C**) Expression of HIF-1α mRNA in samples from each group with mild hypoxia-ischemia, F (DFn, DFd) are F (4, 35) = 231.8. (**D**) Expression of HIF-1α mRNA in samples from each group with severe hypoxia-ischemia, F (DFn, DFd) are F (4, 35) = 361.4. **The difference was statistically significant (*P*<0.01) when compared with the control group.

The 2^−ΔΔCt^ method was applied to compare the expression levels of miRNA-210 in each group of hypoxic-ischemic samples with those in the control group, and it was found that the expression levels of miRNA-210 in each group of hypoxic-ischemic were higher than those in the control group. In mild hypoxic-ischemic group (Figure 4A), the expression levels gradually increased with increasing in the reperfusion time and reached the highest level after 30 min of reperfusion, and then decreased after 1 h of reperfusion, which may be as a results of the repairing process started after the hypoxic insult. While the expression levels decreased after 30 min of reperfusion but the expression level still higher than that in both the control and 10 min group, indicating that the repairing process was not sufficient and the tissues damage still in process. Similar results have been found in the severe group (Figure 4B), where the expression levels reached its peaks after 30 min of reperfusion. Notably, the expression levels in samples in the severe group are higher than those in the mild group. For example, the miR-210 highest expression levels in the mild group after 30 min of reperfusion are (10.639 ± 2.45), while the expression levels in the severe group after the same period of reperfusion are (16.73 ± 3.3), indicating that the tissue damage increased with the hypoxia severity.

In order to understand the mechanism by which miR-210 involve in the hypoxia process, we next sought to investigate the role of HIF-1α mRNA. As expected the expression levels of HIF-1α increased with the reperfusion time and reached the highest level after 30 min of reperfusion in both the mild and severe groups (Figure 4C,D). Similar to results of the miR-210 expression levels the expression levels of HIF-1α in all the samples in the severe group were much higher than those in the mild group at the same reperfusion times. Suggesting that the higher the hypoxia severity the higher the HIF-1α production, in response the expression levels of the miR-210 increased, confirming that miR-210 is controlled by HIF-1α.

The Ct values of HIF-1α mRNA and U6 were obtained from the amplification curves, and the ΔCt was calculated by the formula (ΔCt = CtmRNA-Ct internal reference), and the expression levels of HIF-1α mRNA in the samples of each hypoxic-ischemic group were compared with those of the control group by applying the 2^-ΔΔCt^ method (Figure 4C,D).

### Western blot detection of HIF-1α and SCN1B protein expression levels in fetal rat brain tissue

**Figure 5:**
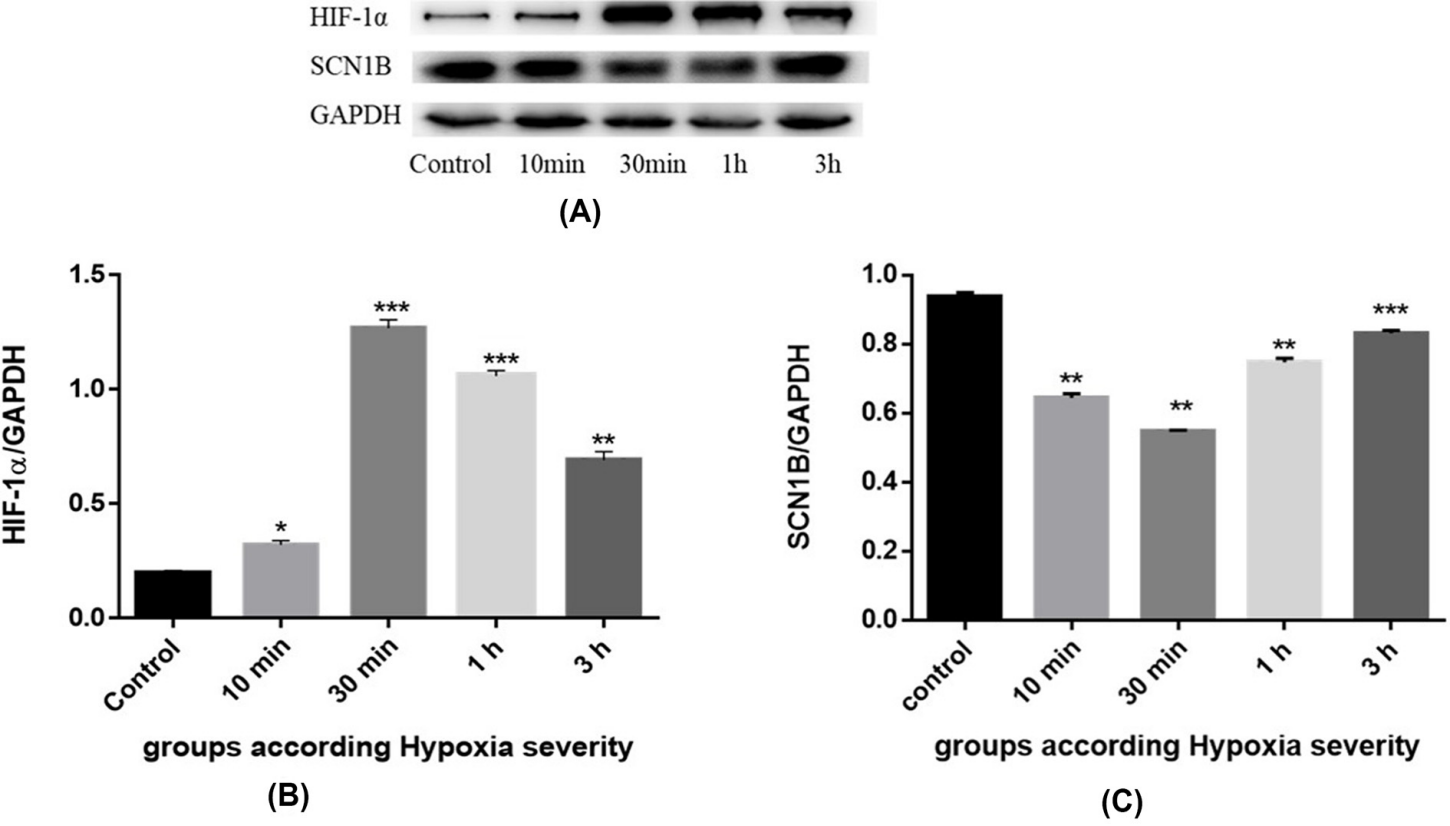
HIF-1α and SCN1B protein levels in rats with brain damage induced by mild hypoxia-ischemia (**A**) HIF-1α and SCN1B protein expression in mild hypoxia-ischemia compared with the control group, F (DFn, DFd) are F (4, 10) = 269.6 and F (4, 10) = 19.630, respectively. (**B**) HIF-1α expression in the brain tissue of fetal rats in the groups with mild hypoxia-ischemia. (**C**) Expression of SCN1B in brain tissue of fetal rats in groups with mild hypoxia-ischemia. **The difference was statistically significant (*P*<0.01) when compared with the control group.

Western blot results showed that the expression of HIF-1α in the control group was lower than that in the mild hypoxic-ischemic group, and the expression levels of HIF-1α reached the highest level after 30 min of reperfusion (Figures 4C and 5A,B). The expression of SCN1B in the control group was higher than that in the hypoxic-ischemic groups, and the expression levels of SCN1B in the mild hypoxic-ischemic with reperfusion 30-min group was the lowest (Figure 5A,C).

**Figure 6:**
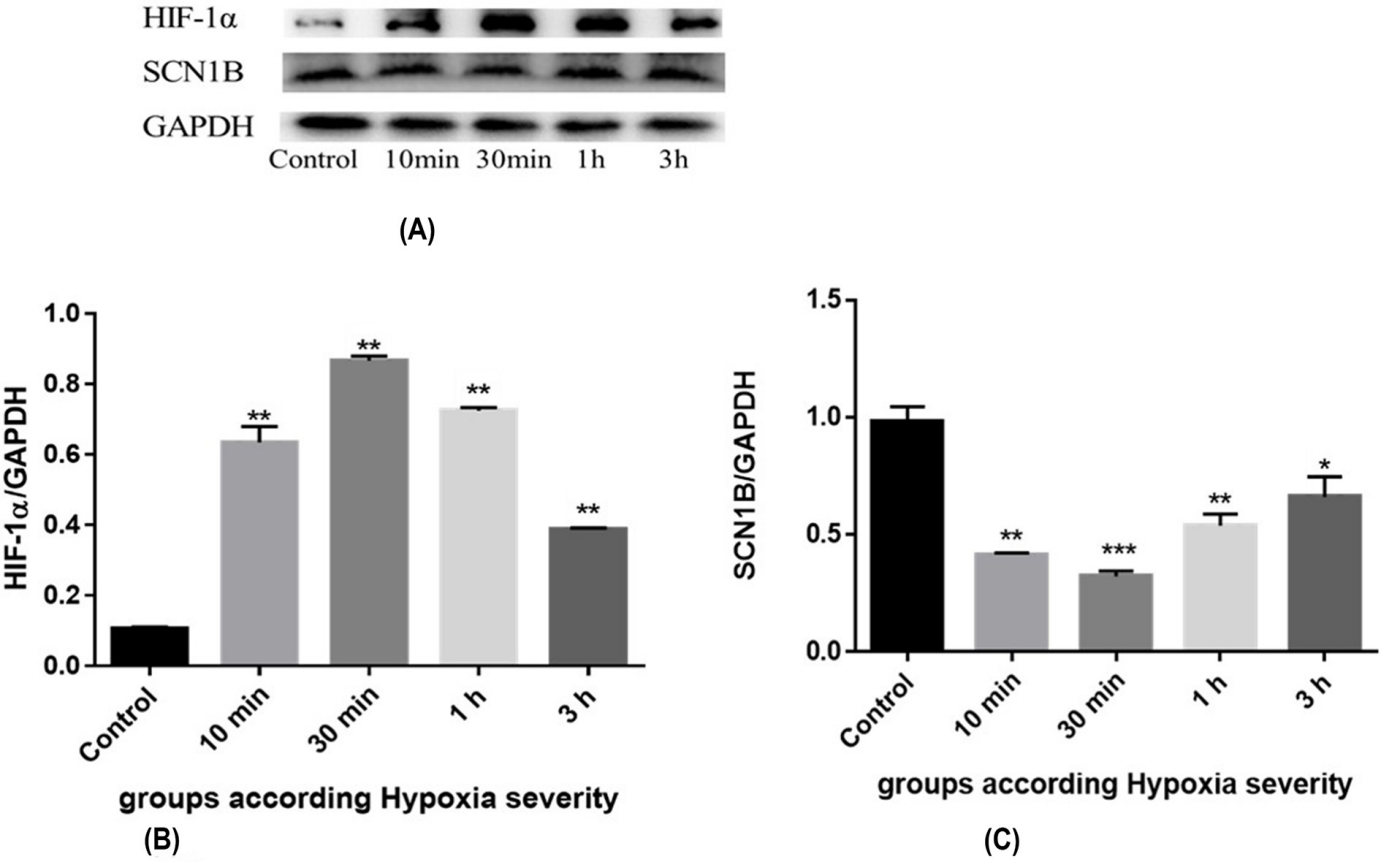
HIF-1α and SCN1B protein levels in rats with brain damage induced by severe hypoxia-ischemia (**A**) HIF-1α and SCN1B protein expression in each group with severe hypoxia-ischemia, F (DFn, DFd) are F (4, 10) = 125.1, and F (4, 10) = 100.1 respectively. (**B**) HIF-1α expression in the brain tissue of fetal rats in each group with severe hypoxia-ischemia. (**C**) Expression of SCN1B in brain tissue of fetal rats in each group with severe hypoxia-ischemia. **The difference was statistically significant (*P*<0.01) when compared with the control group.

Similar to the results found by PCR, we found that the expression levels of HIF-1α in the control group were lower than that in the groups with severe hypoxia-ischemia, and the highest expression levels of HIF-1α were found at 30 min of reperfusion (Figures 4D and 6A,B). While the expression levels of SCN1B in the control group were higher than that in the hypoxic-ischemic groups, and the lowest SCN1B expression levels in the group with severe hypoxia-ischemia were found at 30-min of reperfusion (severe group) (Figure 6 A,C). Indicating that, in response to hypoxia-ischemia the HIF-1α levels increase rising miR-210 expression levels, which in turn decrease the SCN1B levels.

## Discussion

Sodium channels are widely distributed in all tissues of the body and play an important role in maintaining normal physiological functions. The β subunit of sodium channels has various auxiliary functions, including regulating the opening and closing of Na^+^ channels, regulating their distribution and number in cell membranes, interacting with the extracellular matrix as intercellular adhesion molecules, and participating in the regulation of cell migration and aggregation.

Thus, we hypothesize that there may be a link between miRNAs and sodium channel genes, and certain specific miRNAs may affect the expression of ion channel genes, and these specific miRNAs are involved in the HIE process by regulating ion channel genes. Therefore, identifying these miRNAs that can regulate the expression of ion channel genes, and thus using them as new targets to improve hypoxic-ischemic brain injury, is important for HIE. The prevention and treatment of HIE is of great importance.

### Target gene prediction and animal model identification

Bioinformatics is widely used in the prediction of miRNA target genes, which is one of the main tools for miRNA action target research. Some famous bioinformatics websites use high-throughput sequencing technology for miRNA target gene prediction. In the present study, all miRNAs that can bind to SCN1B were predicted by the miRNA target gene prediction websites.

The bioinformatics of miRNA-210 and SCN1B enabled us to better understand their molecular morphology and molecular structure. The molecular structure of miRNA-210 studied by using bioinformatics technology can show the sequence and molecular structure, and the formation process of miRNA can be shown clearly. Bioinformatics can predict gene loci, rapidly screen genes, find relationships between known genes, explore unknown gene functions and other advantages through artificial intelligence methods such as computer software, and the accuracy of prediction is guaranteed through experiments such as gene analysis, protein blotting, microarray, and high-throughput sequencing technologies for validation. Therefore, bioinformatics has become an efficient research tool for more researchers to engage in scientific research. We found the binding site of miRNA-210 in the 3′-UTR region of SCN1B and confirmed that SCN1B is the target gene of miRNA-210 action. MiRNA-210 was finally selected as the target of the study to investigate the mechanism of miRNA-210’s role in hypoxic-ischemic brain injury through regulating SCN1B by combining with the previous research base of the project team.

Animal models of human diseases occupy an important position in the study of the etiology, pathogenesis, and prevention of human diseases. We made an animal model of hypoxic-ischemic brain injury and analyzed the histopathological changes of brain in fetal rats by H&E staining method. The degree of brain tissue damage in the fetal rats was greater in the 30-min reperfusion group than in the 10-min reperfusion group, showing that the degree of brain tissue damage in fetal rats increased with the prolongation of hypoxia-ischemia time.

### Role of HIF-1α and miRNA-210 in HIE

MiRNAs are a class of small noncoding single-stranded RNAs, approximately 19–25 nucleotides in length, that can inhibit the expression of target genes by directly binding to their mRNAs. MiRNAs are first transcribed as precursor molecules and subsequently cleaved by the ribonucleic acid endonucleases Drosha and Dicer to become mature miRNAs. Mature miRNAs bind to Argonaute (AGO) protein family members to form RISC and finally the RNA-induced silencing complex (miRISC) [14]. Recent studies have shown that regulatory molecules can not only regulate the processing steps of individual miRNAs but also link miRNA biosynthesis to other cellular signaling processes. For example, protein phosphorylation links miRNA biosynthesis to various signaling pathways, and such modifications are often associated with disease. Furthermore, not all miRNAs follow classical synthetic routes, and many nonclassical miRNA biosynthetic pathways have recently been identified.

Many studies have shown a direct relationship between hypoxia and miRNA-210 expression in normal and transformed cells, specifically, miRNA-210 expression is up-regulated under hypoxic conditions. Under hypoxic conditions, miRNA-210 is highly up-regulated in cells, revealing its significance for cellular tolerance [15]. Kelly et al. identified a novel HIF-1α regulator, called glycerol-3-phosphate dehydrogenase 1 (GPD1L), which is regulated by HIF-1α-induced miRNA-210. Stimulation of miRNA-210 by HIF-1α induces a significant decrease in GPD1L protein expression, leading to increased HIF-1α stabilization. Under normal biological conditions, GPD1L increases the activity of proline hydroxylase domain isoforms (PHDs) and hydrolyzes HIF-1-proline, ultimately contributing to the degradation of HIF-1α via the proteasome. MiRNA-210 overexpression increases the accumulation of HIF-1α under hypoxic conditions as miRNA binds to the GPD1L mRNA 3′-UTR, which resulting in decreased GPD1L protein expression. In the presence of low HIF-1α protein expression, a decrease in miRNA-210 levels and the resulting up-regulation of GPD1L expression has been observed [16].

Here, we have investigated the role of HIF-1α and miR-210 in regulating SCN1B under the hypoxic conditions. Hypoxic insult resulted in increased HIF-1α expression levels in both the mild and severe groups (Figures 3–6). The increased levels followed by a lagging increase in the miR-210 concentrations in all the groups (Figure 4). The miR-210 is one of the most prominent miRNA stimulated under hypoxic condition. The HIF-1 involves in the regulation process of miR-210 [17]. Our findings demonstrated that, under hypoxic conditions, miR-210 is controlled by HIF-1α.

A decrease in oxygen levels increases HIF-1α protein and gene expression activity, which leads to the accumulation of miRNA-210. GPD1L expression is thus down-regulated and inactivates PHDs, which in turn leads to an increase in HIF-1α protein. The above process consists of an exacerbated feedback loop in which miRNA-210 positively regulates the amount of HIF-1α protein. Inhibition of miRNA-210 can affect this hypoxic cycle. Earlier studies have shown that HIF-2α is involved in the regulation of miRNA-210 [18]. In rats with mild hypoxia-ischemia, the decrease in the oxygen level led to changes in the expression levels of HIF-1α both in blood and brain samples, the degree of changes among samples is somewhat high and depend on the oxygen fellow; at first (reperfusion for 10 min), the expression levels ranging between 9.63 ± 1.21 and 25.47 ± 1.29 ng/ml and with the extension of the reperfusion time (for 30 min) increased again and the expression levels among samples ranging between 10.59 ± 1.52 and 30.66 ± 1.36 ng/ml compared with the control group and reperfusion for less time (10 min) confirming that the HIF-1α levels depend on the oxygen levels. But with extending the reperfusion time again for 1 and 3 h, the expression levels decreased gradually with increasing the reperfusion time. To confirm those results, we sought to do the process again and measure the expression levels in a severe hypoxia group at the same time points (10, 30 min, 1, 3 h). Similar as in the mild group, the expression levels of HIF-1α increased with the decrease in the oxygen levels, and reached its highest level after 30 min then decreased gradually with increasing the reperfusion time. Notably, the HIF-1α levels in the severe group—in which the oxygen fellow is much lower in the mild group in each time point—are much higher when comparing them with that in the mild group at each time point. Altogether, those findings confirm that HIF-1α depends on the oxygen levels in the cell. The decrease in the expression levels suggests that very low oxygen levels lead to tissue damage in which the cells are not able to produce more molecules to repair the damaged organelles.

In the present study, we found that the expression levels of miRNA-210 were higher in all groups of hypoxia-ischemia than in the control group, and the expression was higher in the severe hypoxia-ischemia group (30 min) than in the mild group (10 min), and the highest expression was shown in both the mild and severe groups at 30 min of reperfusion. The above results indicated that the expression of miRNA-210 in the brain started to increase when hypoxia-ischemia occurred and reached the highest level at 30 min of reperfusion that increase is response from the cell in order to control the situation and prevent the ingoing tissue damage due to decrease in oxygen levels, but the expression of miRNA-210 gradually decreased with the extension of reperfusion time, which might be related to the brain tissue damage.

The results of the present study showed that the expression levels of mRNA and protein of HIF-1α were higher in all groups of hypoxia-ischemia than in the control group, and were higher in the group with severe hypoxia-ischemia (30 min) than in the group with mild Hypoxia (10 min), with the highest at 30 min of reperfusion. The change trend of HIF-1α expression was synchronized with miRNA-210, indicating that HIF-1α induced the production of miRNA-210. Altogether, these data confirm that under hypoxia, miRNA-210 is a HIF-1α-dependent miRNA, which is induced by HIF-1α.

### Role of miRNA-210 and SCN1B in HIE

In the present study, we found that the expression level of SCN1B in all groups of hypoxia-ischemia was lower than that in the control group, and the expression level of SCN1B in the severe group of hypoxia-ischemia (30 min) was lower than that in the mild group (10 min), with the lowest at 30 min of reperfusion. The above results indicated that the expression of SCN1B in the brain began to decrease when hypoxia-ischemia occurred, influenced by the change of miRNA-210 expression, and reached the lowest at 30 min of reperfusion. With the extension of reperfusion time, the expression of miRNA-210 gradually increased, which indicated that the repair ability of brain tissue damage is highly affected with the extension of reperfusion time, making the expression to show a trend of first increase and then decrease. The decrease in oxygen levels leads to an increase in HIF-1α, increasing miR-210 expression levels, which in turn controls the SCN1B expression. The increase in miR-210, the decrease in SCN1B levels.

Reduced SCN1B protein expression leads to loss of sodium channel function, and the reduced stimulation and enhanced inhibition caused by reduced sodium current also slows recovery from sodium channel inactivation. β subunit is a transmembrane fragment with an extracellular IgG-like extracellular collateral loop and an intracellular C-terminus. Abnormalities in SCN1B also affect SCN1A function. Mutations in the SCN1A gene encoding the NaV1.1 isoform of the voltage-gated sodium channel can alter the electrophysiological function of the sodium channel, such as shifts in the current–voltage curve, increases in current amplitude, and slower inactivation, resulting in increased excitability. This results in increased excitability of neurons, causing abnormal high-frequency and high-amplitude discharge waves to occur in neurons, which in turn leads to a variety of epileptic pathologies such as hereditary epilepsy with febrile convulsion add-on, DS, and temporal lobe epilepsy.

In summary, miRNA-210 expression was found to be higher in the case group than in the control group, indicating that miRNA-210 expression was increased after cerebral hypoxia-ischemia. Predictions using several bioinformatics databases, such as microRNA-target interaction database, revealed that miRNA-210 had binding sites with SCN1B, suggesting that miRNA-210 inhibited SCN1B protein expression by binding to the SCN1B 3′-UTR region later, resulting in a decrease in SCN1B expression. These results suggest that miRNA-210 can participate in the HIE process by regulating SCN1B expression, and miRNA-210, SCN1B plays an important role in neurological diseases. Based on the above results, it can be speculated that the increase of HIF-1α expression after hypoxia-ischemia induced the increase of miRNA-210 expression, and miRNA-210 targeted to inhibit the expression of target genes, which led to the decrease of SCN1B expression and caused the decrease of SCN1B protein function, which in turn affected the normal function of ion channels and appeared abnormal discharge and other clinical symptoms such as abnormal muscle tone, convulsions, and even epilepsy. This can lead to abnormalities in muscle tone, convulsions, and even epilepsy. MiRNA-210 plays an important regulatory role in HIE by regulating SCN1B expression.

## Data Availability

All data generated or analyzed during the present study are included in the present article.

## Abbreviations

DEPC: diethyl pyrocarbonate water
DS: Dravet syndrome
GPD1L: glycerol-3-phosphate dehydrogenase 1
HIE: hypoxic-ischemic encephalopathy
HRE: hypoxia response element
H&E: haemotoxylin and eosin
PHD: proline hydroxylase domain isoform
SD rat: Sprague Dawley rat
SE: standard error
